# Push-out bond strength for bioceramic and epoxy resin-based sealers

**DOI:** 10.6026/973206300210353

**Published:** 2025-03-31

**Authors:** Jasmine Marwaha, Dipika yadav, Swati Sharma, Ankita Dixit, Ashtha Arya, Sunil kar

**Affiliations:** 1 Department of Conservative Dentistry and Endodontics, Maharishi Markandeshwar College of Dental Sciences and Research, Mullana-133203, India; 2 Department of Dentistry, Maa Vindhyavasini Autonomous State Medical College, Mirzapur-231001, India; 3 Department of Conservative Dentistry and Endodontics, Yogita Dental College and Hospital, Khed, Maharashtra-415709, India; 4 Department of Pedodontics and Preventive Dentistry, Government dental college and hospital, Jamnagar, Gujarat-36100, India; 5 Department of Conservative Dentistry and Endodontics, SGT Dental College, Hospital and Research Institute, SGT University, Gurugram, Haryana- 122505, India; 6 Department of prosthodontics, Research scholar, Institute of dental science SOA university, Ex professor at Hitech dental college and Director of 3S sent care, Bhubaneswar, Odisha-751010, India

**Keywords:** Bio ceramic sealer, epoxy resin-based sealer, push-out bond strength, root canal obturation, endodontics

## Abstract

The push-out bond strength between bioceramic and epoxy resin-based cement materials that treat endodontically treated teeth is of interest to dentist. The push-out bond strength tests were applied to forty premolars after instrumenting and obturating them with either bioceramic or epoxy resin-based sealer materials. The bond strength measurements during the push-out test indicated epoxy resin-based sealer achieved 6.4 ± 0.6 MPa while the bioceramic sealer produced 5.2 ± 0.8 MPa with p < 0.05 indicating substantial statistical difference. The main failure mode in samples with bioceramic sealer involved adhesive breakdown whereas epoxy resin-based sealers demonstrated both cohesive and mixed failure patterns. The bond strength results indicate epoxy resin-based products outperform other products in both adhesion and mechanical interlocking properties which merits deeper examination about their extended clinical utilization.

## Background:

Root canal treatment success depends heavily on endodontic sealers since they provide complete sealing of the system to prevent microbial leakage [1]. The push-out bond strength measurement of these sealers directly affects the retention of root canal fillings after functional loads apply [2]. The clinical practice uses epoxy resin-based and bioceramic-based formulations as sealers because they demonstrate sealing properties along with dentinal biocompatibility alongside bond capability [3, 4]. AH Plus epoxy resin-based sealers demonstrate popular use because they show better adhesion features and minimal solubility while effectively entering dentinal tubules [5]. These dental sealers demonstrate outstanding strength properties because they chemically attach to collagen fibers found in dentin material [6]. The popularity of Endo Sequence BC Sealer increased because it demonstrates bioactive features when releasing calcium ions while creating hydroxyapatite-like structures at the sealer-dentin interface [7, 8]. The lower strength bonds of epoxy resin-based sealers compared to epoxy resin-based sealers may lead to reduced long-term retention within root canals [9]. Research findings about bond strength between these sealers exist in contradiction to one another. Research findings about epoxy resin-based sealer bond strength outcomes vary between distinct studies [10] in comparison to bioceramic sealer advantages in biomineralization and long-term durability [11]. Therefore, it is of interest to examine bioceramic and epoxy resin-based sealer push-out bond strength through universal testing machine analysis.

## Materials and Methods:

This research utilized forty human premolars that had single rooted structures extracted from patients. The research excluded teeth with fractures or previous endodontic treatment and cracked teeth. The researchers preserved the selected samples through storage within distilled water before their eventual use. Rephrased under water cooling a high-speed diamond bur create standardized access cavities. A depth to which the #10 K-file penetrated into the foramen to be seen was set and 1 mm additional length was subtracted from the recorded value.

The authors used ProTaper Universal rotary Ni-Ti files (Dentsply Maillefer) to instrument the canals up to F4 size while irrigating with 5.25% NaOCl and finishing with 17% EDTA solution to eliminate the smear layer. The drying process using paper points was followed by random distribution of twenty samples into group one and group two according to sealer type:

[1] Group 1: Bioceramic sealer (Endo Sequence BC Sealer, Brasseler USA)

[2] Group 2: Epoxy resin-based sealer (AH Plus, Dentsply DeTrey)

The single-cone technique served as the method for obturation while using an F4 gutta-percha cone that matched the size of the final instrumentation. The responsible laboratory experimented with both materials by mixing them per the manufacturer guidelines and then applied them to canal walls with a lentulo spiral while filling with gutta-percha. Workers removed extra materials from the system before the final section received a temporary filling material. The testing environment consisted of 37°C temperature with complete humidity while the samples remained stored for seven days to promote complete sealer setting.

Each prepared root got sectioned into two millimetre thick middle-root parts after the waiting period through diamond tool cutting under water cooling. The measurements of each slice's thickness were confirmed by operating a digital calliper. The universal testing machine (Instron Corp., USA) operated at 0.5 mm/min speed measured the push-out bond strength of the specimens. Filler dislodgement required application of force through an apical-to-coronal direction using a plunger made from stainless steel that was one size smaller than the canal. The lab tested dislodgement force resulted in Newtons (N) measurements which then became megapascals (MPa) by dividing force values by bonded surface area (A = π x r x h, where r is the canal radius and h is the slice thickness). The stereomicroscope at 40x magnification allowed researchers to observe the failure mode of the tested specimens following the push-out test. Experts categorized the observed failure types into three groups.

[1] Adhesive failure: Between sealer and dentin

[2] Cohesive failure: Within the sealer itself

[3] Mixed failure: Combination of adhesive and cohesive

Researchers analyzed the acquired data through SPSS software which IBM Corp. provided from the USA. Statistical evaluation of push-out bond strength values between groups took place by performing an independent t-test. A results of p-value lower than 0.05 was used to establish statistical significance.

## Results:

Push-out bond strength measurements for both sealers appeared as the metric megapascals (MPa). The bioceramic sealer group achieved 5.2 ± 0.8 MPa mean push-out bond strength whereas the epoxy resin-based sealer achieved 6.4 ± 0.6 MPa as its mean bond strength. The data demonstrated a statistically important distinction between groups according to an independent t-test (p < 0.05) as shown in (Table 1). A stereomicroscope revealed different types of failures between the two sealer groups. The bioceramic sealer orientation resulted in 65% adhesive failures while cohesive failures made up 20% and mixed failures composed 15% of the total test cases. The epoxy resin-based sealer tests demonstrated 40% adhesive failures with 30% cohesive failures and 30% mixed failure pattern (Table 2).

The results indicate that the epoxy resin-based sealer exhibited superior bond strength compared to the bioceramic sealer, with a higher incidence of cohesive and mixed failures, suggesting better sealer adaptation and mechanical interlocking (Table 2).

## Discussion:

Endodontic sealer strength during dentin bond formation determines both treatment longevity and the prevention of microleakage and stable obturation material retention [1]. A research analysis revealed that epoxy resin-based sealer demonstrated greater push-out bond strength than bioceramic sealer. Epoxy resin-based sealer achieves superior bond strength through its penetration into dentinal tubes and covalent bond formation with collagen fibrils in dentin matrix [2, 3]. Extensive research shows that AH Plus and other epoxy resin-based sealers demonstrate excellent mechanical properties, low solubility and strong dentinal adhesive capabilities [4, 5]. The chemical bond formation between epoxy resin-based sealer molecules and dentin and their excellent polymerization properties lead to their exceptional bond strength [6]. Research has demonstrated that bioceramic sealers produced weaker bond strength than their bioactive ability which enables hydroxyapatite formation. The sealer-dentin interface bond formation relies on two processes which include chemical development of calcium phosphate compounds and micromechanical interlocking [7, 8]. The push-out bond strength of bioceramic-based sealers falls behind epoxy resin-based sealers because bioceramic materials create more setting expansion along with reduced dentinal tubule infiltration [9, 10]. Previous studies support the higher bond strength values of epoxy resin-based sealers when compared to bioceramic-based sealers [11, 12]. Sealing efficiency studies indicate bioceramic agents excel over time in bond retention since they fuse with dentin whereas they demonstrate weaker initial bond performance [13, 14]. Sealer thickness as well as root canal irrigation methods and obturation techniques individually determine the push-out bond strength values [15]. The bioceramic sealer exhibited primary adhesive failures as the main cause of failure but epoxy resin-based sealer showed more cohesive and mixed failure patterns. The weak bond properties between bioceramic sealers and dentinal tissue create more opportunity for adhesive failure to occur according to research [6]. The epoxy resin-based sealer group demonstrated better internal bond strength characterizing through higher cohesive and mixed failure rates between its components [7]. Clinical sealer selection requires evaluation of both epoxy resin-based sealers and their impact on strength together with biocompatibility and antimicrobial properties as well as long-term dimensional stability [8, 9]. The advanced biocompatibility features combined with periapical healing activation potential of bioceramic-based sealers recommend their usage in procedures seeking improved bioactivity [2]. The research design primarily conducted in test tubes cannot duplicate actual clinical situations because it fails to mimic authentic patient treatments. The bond strength outcomes are affected by variations in dentin composition together with differences in canal morphology and variations in operator techniques [1]. Additional research which combines long-term clinical follow-ups and direct biological investigations must validate the acquired results to establish their medical usefulness [2]. Research studies analyzed root canal sealer bond strength and dentinal tubule penetration along with the adhesion of root canal sealer as it relates to different endodontic irrigants. Experimental results demonstrate that bond strength increases best whenever sealer materials combine with glycolic and phosphoric acid chelating agents especially when they use epoxy-based chemistry. Bioceramic sealers possess excellent adhesion properties yet this performance changes based on the irrigant used. The removal of the smear layer produces major benefits for dentinal tubule penetration of sealers while particular irrigant products impact the total bonding efficiency [16, 17, 18-19].

## Conclusion:

Epoxy resin sealing agents produced superior push-out bond strength measurements relative to the bond strength capabilities of bioceramic sealing agents making them better at attaching to dentin. The adhesive failure seen predominantly in bioceramic groups indicated poor adhesion strength while epoxy resin-based sealer showed mixtures of cohesive failures with adhesive failures which indicated stronger internal and bonding properties.

## Figures and Tables

**Table 1:** Push-out bond strength of bioceramic and epoxy resin-based sealers

**Sealer Type**	**Mean Bond Strength (MPa) ± SD**	**p-value**
Bioceramic Sealer	5.2 ± 0.8	< 0.05*
Epoxy Resin-Based Sealer	6.4 ± 0.6	
*Significant difference between groups (p < 0.05).

**Table 2:** Failure mode distribution among the groups

**Sealer Type**	**Adhesive Failure (%)**	**Cohesive Failure (%)**	**Mixed Failure (%)**
Bioceramic Sealer	65	20	15
Epoxy Resin-Based Sealer	40	30	30
